# Effects of Interleukin-6 (IL-6) on In Vitro Cultured Equine Chorionic Girdle Cells

**DOI:** 10.3390/ani15030450

**Published:** 2025-02-06

**Authors:** Siqin Mu, Yingchao Shen, Hong Ren, Tseweendolmaa Ulaangerel, Minna Yi, Bilig Zhao, Asiya Hao, Qi Liu, Xin Wen, Manglai Dugarjaviin, Gerelchimeg Bou

**Affiliations:** Inner Mongolia Key Laboratory of Equine Science Research and Technology Innovation, Inner Mongolia Agricultural University, Hohhot 010018, China; siqin20220919@163.com (S.M.); shenyingchao2017@163.com (Y.S.); renhong1980@126.com (H.R.); cewendclma@126.com (T.U.); yiminna2020@163.com (M.Y.); bilig9@163.com (B.Z.); 15124926900@163.com (A.H.); liuqi99967@163.com (Q.L.); wenxin618@imau.edu.cn (X.W.); dmanglai@163.com (M.D.)

**Keywords:** equine, chorionic girdle cells, equine chorionic gonadotrophin, interleukin-6, invasion

## Abstract

Introduction: Equine chorionic girdle cells can give rise to the endometrial cup, a structure that secretes equine chorionic gonadotropin (eCG). To date, the mechanisms underlying the proliferation, differentiation, invasion, and hormone secretion of equine chorionic girdle cells are not fully understood. During human pregnancy, interleukin-6 (IL-6) is maternally expressed to stimulate the invasion and migration of human syncytiotrophoblast cells and influence the synthesis of human chorionic gonadotropin. Although many previous studies reported the same upregulation of IL-6 during equine placentation, its effects on equine chorionic girdle cells have never been tested. Methods: In this study, we tested the effects of different concentrations of IL-6 on the morphology, differentiation, proliferation, hormone production, invasion, migration, and gene expression profiles of equine chorionic girdle cells. Results: We found that, with respect to morphology and proliferation, IL-6 had no significant effect; with respect to eCG production, 30 ng/mL IL-6 significantly increased the expression of genes related to eCG production, whereas treatment with 70 ng/mL IL-6 downregulated these genes. However, no significant increase in intracellular protein levels or eCG secretion was observed following treatment with any concentration of IL-6. Regarding cell migration and invasion, we found that IL-6 treatment had no significant effect on the migration capability of equine chorionic girdle cells but did enhance the invasion capability of equine chorionic girdle cells and upregulated the expression of the invasion-related genes, Mmp2 and Mmp9. Finally, our transcriptomic study revealed that in equine chorionic girdle cells, IL-6 treatment mainly affected the expression of genes related to the NOD-like receptor signaling pathway and the JAK–STAT signaling pathways, which are involved in immune and inflammatory responses. Conclusions: In summary, this study demonstrates the positive effects of IL-6 on the cytokine secretion and invasive ability of equine chorionic girdle cells.

## Simple Summary

This study investigated the effects of IL-6 on equine chorionic girdle cells. IL-6 did not significantly affect cell morphology, proliferation, or eCG secretion. However, 30 ng/mL IL-6 increased eCG-related gene expression, while 70 ng/mL IL-6 reduced it. IL-6 enhanced cell invasion and upregulated invasion-related genes (MMP2, MMP9), but did not impact migration. Transcriptomic analysis showed that IL-6 influenced genes in the NOD-like receptor and JAK–STAT pathways, which are involved in immune and inflammatory responses. Overall, IL-6 positively affected cytokine secretion and invasiveness in these cells.

## 1. Introduction

Equine chorionic gonadotropin (eCG), also known as pregnant mare serum gonadotropin (PMSG), was first discovered in the serum of pregnant mares by Harold Cole and George Hart in 1930 [1]. eCG exhibits dual activity similar to that of follicle-stimulating hormone (FSH) and luteinizing hormone (LH), but only the LH effect in mares, while the FSH effect is only obvious in non-equine animals. eCG has been widely applied in livestock production and veterinary clinical practice [2,3,4]. At present, the main source of eCG is the whole blood of pregnant mares, which seriously affects the welfare of mares and may lead to a large number of fetal deaths [5]. Therefore, it is necessary to establish a large number of commercial in vitro systems for stable eCG production to protect the welfare of equine animals.

eCG is secreted by the endometrial cup tissue of pregnant mares, which originates from fetal chorionic girdle cells that rapidly proliferate, differentiate, and invade the endometrium around days 30–40 of pregnancy. During this period, these cells begin to secrete high concentrations of eCG [6,7,8,9,10,11]. Research has revealed that various growth factors, including hepatocyte growth factor-scatter factor (HGF-SF), epidermal growth factor (EGF), vascular endothelial growth factor (VEGF), and transforming growth factor-beta (TGF-β), play roles in the process of mare pregnancy [12,13,14,15]. However, the regulatory mechanisms controlling the formation and terminal differentiation of equine chorionic girdle cells, invasion into the endometrium to form endometrial cups, and secretion of eCG remain unclear.

IL-6 is a multifunctional cytokine and a member of the cytokine family involved in various physiological processes, such as organ development, the acute phase response, inflammation, the immune response, and metabolic regulation [16,17,18]. IL-6 exerts its effects by binding to its receptor and activating the JAK–STAT pathway [19,20]. There are two signaling mechanisms for IL-6: classic signaling and trans-signaling. Its proinflammatory effects are primarily mediated through IL-6/sIL-6R trans-signaling, while its anti-inflammatory effects are mainly mediated by classic IL-6R signaling [21,22]. Studies have shown that mares produce IL-6 during pregnancy and that fetal-derived IL-6 may have anti-inflammatory effects [23,24].

Human chorionic gonadotropin (hCG) and equine chorionic gonadotropin are placental hormones that are specific to humans and horses. hCG is secreted by human syncytiotrophoblast cells, which originate from human cytotrophoblast cells (CTBs), analogous to chorionic girdle cells in equines. Studies indicate that IL-6 can stimulate trophoblast invasion and migration in primary early pregnancy CTB and EVT cell lines, such as HTR-8/SVneo, by increasing integrin α5, α1, and β1 expression in syncytiotrophoblasts and activating MMP-2 and MMP-9 to mediate this process [25,26,27]. Additionally, IL-6 plays a crucial role in spiral artery remodeling and is considered a key factor derived from EVTs that activate endothelial cells to release chemokines for dNK cells and macrophages, thus initiating spiral artery remodeling [28]. IL-6 has also been shown to modulate the immune–endocrine crosstalk during pregnancy, influencing the synthesis of human chorionic gonadotropin and the human placental lactogen β subunit [29,30]. The spatial and temporal distributions of IL-6 and its corresponding receptors clearly indicate its significant role in early human pregnancy events.

This study aimed to investigate the effects of IL-6 on eCG secretion and the invasive ability of equine chorionic girdle cells. To study the effects of different concentrations of IL-6 on equine chorionic girdle cells, transcriptome sequencing was used to analyze its molecular regulatory pattern comprehensively. This study provides a theoretical foundation for understanding the differentiation, invasion, and eCG secretion mechanisms of equine chorionic girdle cells.

## 2. Materials and Methods

### 2.1. Culture and Stimulation of Chorionic Girdle Cells

The cells used in this study were derived from equine chorionic girdle cells isolated and cultured in the laboratory from 30-day-gestation Mongolian horse embryos [31]. Cells were removed from liquid nitrogen and rapidly thawed at 37 °C to resuspend in DMEM-F12 medium containing 100 U/mL penicillin, 100 U/mL streptomycin, 10 μM epidermal growth factor, and 10% FBS (Gibco, Grand Island, NY, USA). The cells were cultured at 37 °C under 5% CO_2_. For experimental treatments, recombinant IL-6 (MCE, Monmouth Junction, NJ, USA) was added to the cells at different concentrations (10, 20, 30, 50, or 70 ng/mL), while cells without IL-6 (0 ng/mL) served as the control group. After 48 h of treatment, the cells and culture media were collected and stored at −20 °C or −80 °C.

### 2.2. Cell Proliferation Detected by the CCK-8 Assay

Equine chorionic girdle cells cultured to 90% confluence were collected via 0.25% trypsin–EDTA, and approximately 5 × 10^3^ cells were seeded into each well of a 96-well plate. After cell adhesion, the experimental groups were switched to a complete culture medium containing different concentrations of IL-6. CCK-8 reagent was added at 24 h, 48 h, 72 h, and 96 h of culture, followed by a 2-h light-protected incubation in the culture chamber, and the absorbance at 450 nm was measured. The data were analyzed and graphed using Excel software and GraphPad Prism 9 software.

### 2.3. RT-qPCR

Commercial kits (RNeasy Mini Kits, QIAGEN, Duesseldorf, Germany) were used to isolate RNA from the samples, and the concentration and purity of the collected RNA were quantified via a spectrophotometer. Following the manufacturer’s protocol, PrimeScript RT Master Mix (TaKaRa, Tokyo, Japan) was used for first-strand cDNA synthesis. RT-qPCR was performed with TB Green^®^ Premix Ex Taq™ II reagent (TaKaRa, Tokyo, Japan) on a CFX96Touch real-time PCR system (Bio-Rad, Hercules, CA, USA). Primers were designed using Primer 5.0 software based on the full-length mRNA sequences of horses downloaded from NCBI GenBank and synthesized by Sangon Biotech (Beijing, China), as shown in Supplementary Table S1. ACTB was used as the reference gene.

### 2.4. Western Blot and ELISA Analysis

The cells were lysed on ice in RIPA protein lysis buffer containing PMSF (Solarbio, Beijing, China). The protein concentration of each sample was determined via a BCA protein quantification kit (Solarbio, Beijing, China). The samples were boiled in 5× protein loading buffer for 5 min, with 30 μg of protein loaded per lane. Prestained protein standards were used as molecular weight markers.

SDS-PAGE was conducted on 10% polyacrylamide gels with 4% stacking gels for 50 min to separate proteins by electrophoresis, followed by transfer onto nitrocellulose membranes. The transfer was performed at a constant voltage of 100 V for approximately 50 min. The membranes were incubated overnight with a 1:1000 dilution of polyclonal antibodies against anti-chorionic gonadotropin (MyBioSource, San Diego, CA, USA) and GAPDH (Affinity Biosciences, OH, USA), all in phosphate-buffered saline-Tween 20 containing 5% skim milk powder.

The membranes were then shaken with a 1:30,000 dilution of anti-rabbit IgG peroxidase secondary antibody in phosphate-buffered saline containing Tween 20 with 5% skim milk powder for 1 h with shaking. Visualization of eCG protein was achieved by incubation with ECL Western blotting Substrate (Promega. Madison, WI, USA) and exposure to Hyperfilm ECL. Grayscale analysis of intensity values was performed using ImageJ (V1.52h) software.

After cultured chorionic gonadotropin cells were treated with different concentrations of IL-6 for 96 h, the culture medium was collected from the cells. The concentration of eCG in the culture medium was determined using the equine chorionic gonadotropin enzyme-linked immunosorbent assay (DRG International, Springfield, NJ, USA), as previously described [32].

### 2.5. Cell Migration and Invasion Assays

The experiment utilized Culture-Insert 2 wells in a 35 mm µ-Dish (Ibidi, Munich, Germany). Chorionic girdle cells were seeded at 5 × 10^4^ cells/mL into each well of the Culture-Insert 2 Well. The mixture was incubated at 37 °C with 5% CO_2_ until confluence was reached. After the Culture-Insert 2-well was gently removed with sterile forceps, the cell layer was washed with DPBS to remove cell debris and nonadherent cells. Subsequently, cell culture media without or with 10, 20, 30, 50, or 70 ng/mL IL-6 (500 μL) were added. Predetermined areas were photographed at 0 h, 12 h, and 24 h. The area of the exposed regions was measured using ImageJ software, and the results are expressed as a percentage relative to the control.

The transwell invasion assay was conducted in 24-well plates with membranes (pore size 8 μm; NEST, Wuxi, China). Briefly, cells suspended in serum-free medium containing different concentrations of IL-6 (0, 30, and 70 ng/mL) were adjusted to 6 × 10^4^ cells in 200 μL. These cells were then seeded into the upper chamber of Matrigel-coated transwell inserts. The lower chamber contained 500 mL of medium supplemented with 10% FBS. The cells were cultured at 37 °C with 5% CO_2_ for 24 h. The transwell chambers were subsequently washed twice with warm PBS, and a cotton swab was used to gently remove the cells from the upper surface. The cells on the lower surface were fixed in 4% paraformaldehyde for 30 min at room temperature. Chorionic girdled cells were stained with 0.1% crystal violet staining solution. Using an optical microscope (ZEISS, Baden-Württemberg, Germany), images of five nonoverlapping areas of the membrane were taken, and invading cells were counted. The experiment was performed in triplicate, and the results are reported as the number of invading cells.

### 2.6. RNA Sequencing

Compared with the control group, the experimental groups that significantly affected chorionic girdle cells were selected for transcriptomic analysis.

All sequencing work was performed by Annoroad Gene Technology (Beijing, China). The purity, concentration, and RNA integrity of the samples were assessed via the Nanodrop 2000 and Labchip GX Touch systems. The library construction process included mRNA enrichment, fragmentation, cDNA synthesis, end repair, adapter ligation, and selection of appropriately sized cDNA fragments. PCR amplification was subsequently performed to obtain the cDNA library. The concentration and fragment size of the library were initially evaluated via the Qubit^®^ 3.0 and Agilent 2100 systems, followed by precise quantification of the library concentration via the Bio-Rad CFX96 real-time quantitative PCR instrument. Finally, different libraries were pooled and subjected to paired-end sequencing on the Illumina platform with a read length of 150 bp.

### 2.7. Bioinformatics Analysis

Data quality control was first performed, filtering the raw data to remove adapter sequences, sequences containing ‘N’, and low-quality sequences, while the Q30 and GC contents were calculated. The cleaned data were then aligned to the reference genome using HISAT2 software to calculate the FPKM values for each gene, thus allowing the assessment of gene expression levels. Differential expression analysis was conducted using DESeq2 (V1.22.2) software. Finally, cluster Profiler software was used for GO enrichment analysis and KEGG pathway analysis of the DEGs to identify significantly enriched GO terms and KEGG pathways.

### 2.8. Statistical Analysis

The data were analyzed via one-way analysis of variance (ANOVA) to assess the statistical significance of differences between groups. Differences were considered significant when the *p* value was less than 0.05. The values are presented as the means ± standard errors (SEMs), and all the experiments were repeated three times. Groups that do not share a common letter are considered significantly different, whereas those that share the same letter are not significantly different. The uppercase letters denote significance at the *p* < 0.01 level.

## 3. Results

### 3.1. The Impact of IL-6 on Cell Morphology and Proliferation

**Figure 1:**
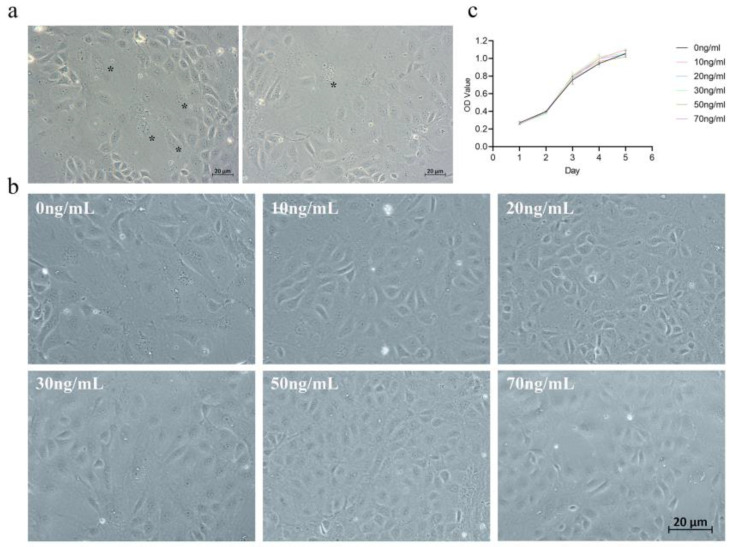
Morphology and proliferation of equine chorionic girdle cells following IL-6 treatment. (**a**) In vitro cultured chorionic girdle cells, with binucleated and multinucleated cells marked with asterisks (*). (**b**) Proliferation curves of chorionic girdle cells treated with different concentrations of IL-6, represented by OD values, showing no significant differences among the groups (*p* > 0.05). (**a**,**b**): 20 µm. (**c**) Morphology of chorionic girdled cells after treatment with different concentrations of IL-6.

The cultured equine chorionic girdle cells mainly consisted of mononuclear trophoblast layer cells and a small number of binuclear cells, where the volume of binuclear cells was notably greater than that of mononuclear cells. Additionally, fused multinuclear cells were observed in in vitro cultured chorionic girdle cells (Figure 1a). Moreover, IL-6 did not significantly affect the proliferation of chorionic girdle cells (Figure 1b). Following the addition of different concentrations of IL-6 to the cell culture medium, no significant changes in cell morphology were observed. Compared with the control, the addition of five different concentrations of IL-6 did not significantly promote the differentiation of mononuclear cells into binuclear cells, nor did it increase the number of binuclear cells (Figure 1c).

### 3.2. The Effect of IL-6 on the Secretion of eCG by Equine Chorionic Girdle Cells

**Figure 2:**
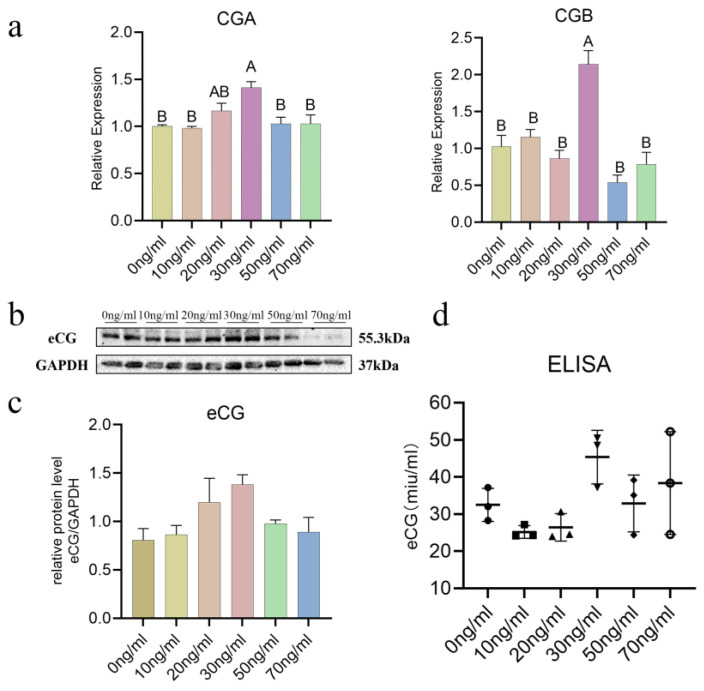
IL-6 has concentration-dependent effects on mRNA (**a**) and protein (**b**,**c**) expression related to eCG secretion and on hormone secretion levels (**d**) in chorionic girdle cells. The uppercase letters denote significance at the *p* < 0.01 level. There is no significant difference between the groups, and the graphs are not marked with letters.

Following the treatment of equine chorionic girdle cells with different concentrations of IL-6 for 48 h, changes in the expression of genes related to the secretion of the eCG subunits CGA and CGB were assessed via RT-qPCR experiments (Figure 2a). The results revealed significant upregulation of CGA and CGB gene expression within 48 h following treatment with 30 ng/mL IL-6 (*p* < 0.01).

Furthermore, within 48 h of IL-6 treatment, a trend toward increased eCG protein expression from the 0 ng/mL group to the 30 ng/mL group was observed, although this difference did not reach statistical significance (*p* > 0.05) (Figure 2b). ELISA analysis of the cell culture supernatant after IL-6 treatment revealed an elevated level of eCG secretion in the 30 ng/mL group compared with the 0 ng/mL group. Similarly, this difference was not statistically significant (*p* > 0.05) (Figure 2c).

### 3.3. The Impact of IL-6 on the Migration and Invasive Capabilities of Equine Chorionic Girdle Cells

**Figure 3:**
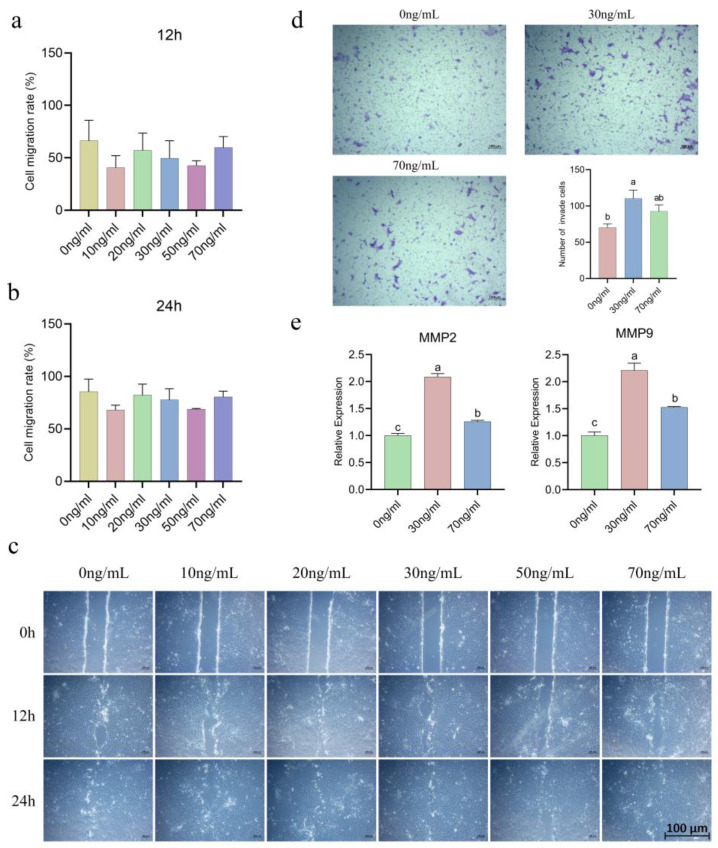
Different concentrations of IL-6 had no significant effect on the migration capacity of chorionic girdled cells (**a**–**c**) but had concentration-dependent effects on their invasive ability (**d**,**e**), *p* < 0.05. (**c**,**d**): 50 µm. Lowercase letters indicate the significance of *p* < 0.05.

The results of the cell migration experiments revealed that treatment with different concentrations of IL-6 for 12 and 24 h did not significantly affect the migration of equine chorionic girdle cells, and there were no significant differences between the groups (*p* > 0.05) (Figure 3a–c).

To explore the role of IL-6 in the invasion of equine chorionic girdle cells, transwell invasion assays were conducted to assess the impact of IL-6 on their invasive ability. The results revealed a significant increase in the number of invasive cells after treatment with 30 ng/mL IL-6 (*p* < 0.05) (Figure 3d). This experiment also measured the mRNA expression levels of the invasion-related genes MMP2 and MMP9, revealing that treatment with 30 ng/mL IL-6 significantly upregulated the expression of MMP2 and MMP9 (Figure 3e), which was consistent with the invasion assay results.

### 3.4. RNA-seq Analysis Revealed the Effects of IL-6 on the Gene Profiles of Equine Chorionic Girdle Cells

The total RNA extracted from equine chorionic girdle cells in this study exhibited good integrity and no contamination upon inspection. The RNA concentrations of the samples were all above 100 ng/μL, with OD260/280 values ranging from 2.1–1 to 2.2. We selected equine chorionic girdle cells without added IL-6 as the control group (samples labeled as CG01, CG02, and CG03) and cells treated with 30 ng/mL and 70 ng/mL IL-6 as the experimental groups (samples labeled as CG31, CG32, CG33, and CG71, CG72, CG73) for transcriptomic library construction and sequencing. After filtering for adapter sequences and other criteria from the raw sequencing data, the statistical analysis of the clean read information revealed that the percentage of Q30 bases was no less than 95% across all the samples, and the total GC content ranged from 52 to 54% (Supplementary Table S2). Both the control and experimental libraries presented ≥98% alignment rates to the genome sequences (Supplementary Table S3). These results indicate that the sequencing data quality of the nine cDNA libraries in this study is robust and suitable for subsequent analyses.

**Figure 4:**
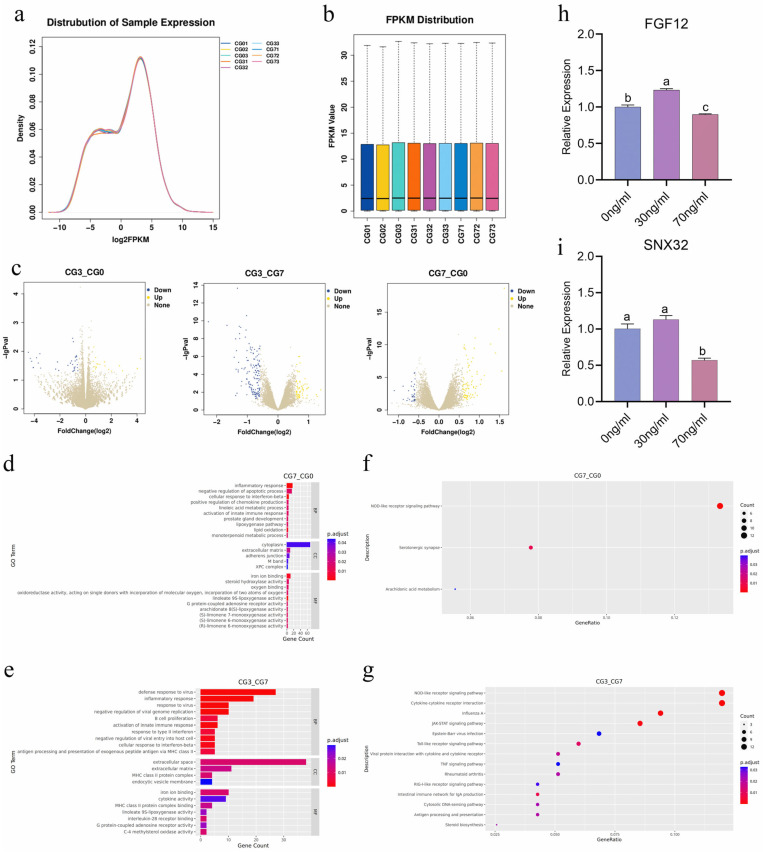
Basic information and verification of transcriptome sequencing data. (**a**) Density distribution plot of gene expression levels across samples. (**b**) Box plot showing gene expression levels across samples. (**c**) Statistical graph of differentially expressed genes. CG01, CG02, CG03: Control group (0 ng/mL IL-6); CG31, CG32, CG33: Experimental group (30 ng/mL IL-6); CG71, CG72, CG73: Control group (70 ng/mL IL-6). (**d**,**e**) Gene Ontology (GO) enrichment analysis of upregulated genes in the experimental group. (**f**,**g**) KEGG pathway enrichment analysis of upregulated genes in the experimental group. (**h**,**i**) RT-qPCR detection of FGF12 and SNX32 gene expression in the 30 ng/mL group. *p* < 0.05.

Comparisons of gene expression density and overall trends among the samples revealed similar distributions of gene expression density (Figure 4a). The FPKM distributions of the gene expression levels were relatively uniform across all the samples (Figure 4b).

In equine chorionic girdle cells treated with 30 ng/mL or 70 ng/mL IL-6 compared with control cells, significantly differentially expressed genes were identified through differential gene expression analysis. Genes were filtered based on the criteria of a fold change ≥ 1.5, *p* value ≤ 0.05, and *p* adj ≤ 1. Compared with the control group (0 ng/mL), the 30 ng/mL group presented 53 differentially expressed genes, including 20 upregulated genes and 33 downregulated genes. Similarly, compared with the control group (0 ng/mL), the 70 ng/mL group presented 281 differentially expressed genes, with 175 genes upregulated and 106 genes downregulated. In contrast, the 30 ng/mL and 70 ng/mL groups collectively presented 342 differentially expressed genes, with 124 genes upregulated and 218 genes downregulated in the 30 ng/mL group compared with the 70 ng/mL group (Figure 4c, Supplementary Table S4–S6).

Transcriptome sequencing analysis revealed that, compared with the control group (0 ng/mL), the 30 ng/mL group had fewer differentially expressed genes, and no significant GO or KEGG enrichment results were observed. In the 70 ng/mL group, GO enrichment analysis of the upregulated genes revealed significant enrichment in biological processes such as the inflammatory response, negative regulation of cell apoptosis, negative regulation of chemokine production, and activation of the innate immune response. At the cellular component level, the enriched terms included extracellular matrix, cytoplasm, and adhesion molecule binding. Molecular function analysis revealed significant enrichment of functions such as iron ion binding and monooxygenase activity (Figure 4d).

Comparing the 30 ng/mL group with the 70 ng/mL group, GO enrichment analysis of upregulated genes in the 30 ng/mL group revealed significant enrichment in biological processes, including the antiviral defense response, inflammatory response, B cell proliferation, activation of the innate immune response, type II interferon response, cell response to interferon-beta, and antigen processing and presentation of MHC class II peptide antigen. At the cellular component level, the enriched terms included extracellular space, extracellular matrix, MHC class II protein complex, and endocytic vesicle membrane. Molecular function analysis revealed significant enrichment of functions such as iron ion binding, cytokine activity, MHC class II protein complex binding, interleukin-28 receptor binding, and G protein-coupled adenosine receptor activity (Figure 4e).

Compared with those in the control group (0 ng/mL), KEGG pathway enrichment analysis of the upregulated genes in the 70 ng/mL group revealed significant enrichment in the NOD-like receptor signaling pathway, serotonin synapse pathway, and arachidonic acid metabolism pathway (Figure 4f). Compared with those in the 70 ng/mL group, KEGG pathway enrichment analysis of the upregulated genes in the 30 ng/mL group revealed significant enrichment in the NOD-like receptor signaling pathway, cytokine–cytokine receptor interaction, JAK–STAT signaling pathway, Toll-like receptor signaling pathway, TNF signaling pathway, and antigen processing and presentation pathway (Figure 4g).

Transcriptomic sequencing analysis revealed that genes with relatively high expression levels in the 30 ng/mL group, such as fibroblast growth factor 12 (FGF12) and sorting Nexin 32 (SNX32), are involved in cell proliferation, migration, adhesion, and protein transport. Validation of these genes via RT-qPCR revealed that their mRNA expression trends were consistent with the transcriptomic sequencing data, but the 30 ng/mL group was significantly higher than the 70 ng/mL group (Figure 4h,i).

## 4. Discussion

Starting around the 30th day of pregnancy, the embryonic chorionic girdle cells begin to proliferate and differentiate into binucleate cells, subsequently invading the endometrium to form endometrial cups, which then commence extensive secretion of eCG [11]. However, to date, there is no definitive conclusion on how these chorionic girdle cells complete this series of processes within the mare’s body.

Observations of chorionic girdle cells cultured in vitro revealed that the majority of the cells appeared as mononuclear epithelioid cells, with only a very small number presenting in a binuclear state, which is consistent with observations by Salman et al. [33]. Our study revealed that the addition of IL-6 to the cells did not significantly alter cell morphology or promote significant differentiation and proliferation. The differentiation of chorionic girdle cells into binucleate cells capable of secreting eCG may require coordinated interactions of various intrinsic and extrinsic factors.

Multiple studies have shown that IL-6 interacts with the IL-6 receptor on the surface of human trophoblast cells [29], thereby promoting the release of human chorionic gonadotropin (hCG) [34,35,36]. In this study, adding IL-6 to equine chorionic girdle cells significantly increased the mRNA expression levels of CGA and CGB. This increase was dose-dependent at low concentrations but significantly inhibited after 48 h of IL-6 treatment at concentrations of 50 and 70 ng/mL. In view of the results observed in other studies [27,29,37], we hypothesize that the peak observed at the concentration of 30 ng/mL IL-6 followed by a decrease may be due to the activation of a negative feedback mechanism in cells at higher IL-6 concentrations, inhibiting its own function. For example, IL-6 can activate the expression of some inhibitory factors, which may reduce the effect of IL-6 through negative feedback mechanism [38]. At the concentration of 70 ng/mL, it may trigger a stronger negative feedback reaction, thus weakening the cell’s response to IL-6. We further examined the levels of eCG secretion and protein expression in equine chorionic girdle cells after the addition of different concentrations of IL-6. Although the differences were not significant, the protein expression levels and eCG secretion in the IL-6 treatment group were generally consistent with the mRNA expression trend, suggesting that IL-6 may play a partial regulatory role in eCG secretion in vivo.

The transcriptome sequencing results of this study indicate that most genes are significantly enriched in biological processes related to immune and inflammatory responses according to the GO enrichment analysis. Additionally, KEGG analysis revealed that these genes are significantly enriched in the NOD-like receptor signaling pathway and the JAK–STAT signaling pathway, with studies revealing that the NOD-like receptor signaling pathway plays a role in pregnancy and innate immunity [39,40]. The JAK–STAT signaling pathway regulates various cellular functions, including proliferation, migration, differentiation, and apoptosis [41], and plays a significant regulatory role in immune functions [42,43,44]. In horses, activation of the JAK–STAT signaling pathway can lead to anti-inflammatory and antiapoptotic effects and promote fetal survival [23]. In this study, we hypothesize that IL-6 has an immunoregulatory effect on equine chorionic girdle cells; however, this hypothesis requires further validation through in vivo and in vitro experiments.

During mare pregnancy at 30 days, chorionic girdle cells display a heightened invasive nature upon receiving signals. Initially, noninvasive cells gradually transform into invasive chorionic girdle cells, infiltrating the epithelium of the uterine endometrial cavity and migrating downward to the glands [45]. This process is crucial for the formation of endometrial cups in mares and successful pregnancy. In other species and pregnant mares, failure of trophoblast cell invasion may lead to various pregnancy complications, such as preeclampsia, fetal growth restriction, premature birth, and miscarriage, posing serious threats to maternal and offspring health [38].

The mechanisms regulating the invasion, movement, and migration of equine chorionic girdle cells are not yet fully understood. In the migration experiments of this study, IL-6 treatment did not significantly affect the migration of chorionic girdle cells, which may be due to the lack of corresponding inhibitory effects on cell proliferation; therefore, differences in cell proliferation after treatment with different concentrations of IL-6 were not detected. However, our invasion experiments revealed that IL-6 significantly enhanced the invasive ability of equine chorionic girdle cells, particularly at a concentration of 30 ng/mL. Although no significant difference was observed at a concentration of 70 ng/mL, the number of invasive cells was still significantly greater than that in the control group. This promoting effect of IL-6 on the invasion of equine chorionic girdle cells is similar to the experimental results in human trophoblast cells [25,46]. Meisser et al. demonstrated that IL-6 can activate MMP-2 and MMP-9 in trophoblast cells (CTB) in vitro [27]. Furthermore, studies have revealed the expression of active MMP-2 and MMP-9 in fetal cells during early pregnancy in mares. Invasive chorionic girdle cells produce MMP-2 and MMP-9 during the establishment of the endometrial cup in the mare’s uterus, indicating that MMPs play important roles in early pregnancy [47], which aligns with our findings in equine chorionic girdle cells.

To validate the accuracy of the transcriptome sequencing results, we selected two genes for RT-qPCR verification: FGF12 and SNX32. FGF12 expression was low in the 0 ng/mL IL-6 group, upregulated in the 30 ng/mL IL-6 group, and subsequently downregulated in the 70 ng/mL IL-6 group. The verification results were consistent with the transcriptome sequencing findings. FGF12 belongs to the fibroblast growth factor (FGF) family, which plays important regulatory roles in cell division, differentiation, and angiogenesis [48,49]. Although the exact role of FGFs in the equine placenta remains unclear, studies have indicated that during the development of chorionic girdle cells, the signaling of these factors may have autocrine functions and exert paracrine effects on adjacent tissues, such as the yolk sac membrane, thereby coordinating the development and implantation of the chorionic girdle [50]. MEGF11, ELFN1, CTNNA2, CHADL, ITM2A, PGLYRP4, and others were also upregulated in the 30 ng/mL IL-6 group, but there are very few reports on the roles of these genes during pregnancy.

Harman et al. reported that, after injecting small pieces of equine chorionic girdle tissue into the backs of severe combined immunodeficiency (SCID) mice, high levels of eCG were detected in the blood compared with those in normal mice. Additionally, the longevity of the grafts was similar to that of the endometrial cup cells in equines, whereas, in normal mice, there was no chorionic girdle engraftment. The lifespan of equine endometrial cup cells is not determined by maternal immune responses but rather by intrinsic factors of the cup cells [51]. This may also partially explain why the protein expression and eCG secretion of equine chorionic girdle cells in this study did not significantly change after the addition of IL-6.

In conclusion, the addition of IL-6 to the culture medium of equine chorionic girdle cells stimulates the expression of eCG and promotes their invasiveness. These findings suggest that IL-6 at the maternal–fetal interface in equids may play a partial role in the invasion of the uterine endometrium by chorionic girdle cells and the secretion of eCG by the endometrial cups, but it does not seem to directly lead to the secretion of eCG in vitro. Riley et al. constructed chorionic girdle organoids from primary chorionic girdle tissue and produced more eCG than monoculture chorionic girdle cells [52]. In the future, key genes or pathways could be identified and applied to organoids to generate stable production models of eCG in vitro.

To date, the molecular mechanisms regulating the differentiation, invasion, and eCG secretion of equine chorionic girdle cells remain unknown. In the future, efforts should focus on applying single-cell transcriptomic methods to further elucidate the developmental patterns of the chorionic girdle in the maternal environment and the molecular pathways regulating hormone secretion. This approach could help reveal the potential role of equine chorionic girdle differentiation and subsequent hormone secretion in embryonic development and healthy fetal growth.

## 5. Conclusions

This study demonstrated that IL-6 treatment has no significant effect on the morphology, proliferation, or migratory ability of equine chorionic girdle cells in vitro. Additionally, it does not have a notable effect on the intracellular protein levels or secretion of eCG. However, IL-6 affects the in vitro invasiveness and eCG transcription levels of chorionic girdle cells by regulating relevant signaling pathways and gene expression at different concentrations. This study suggests that blood IL-6 levels in day 30 pregnant mares could potentially serve as a marker for successful embryo implantation and trophoblast invasion.

## Data Availability

All data are accessible through the article and its Supplementary Materials file or upon request from the authors.

## Supplementary Materials

The following supporting information can be downloaded at: https://www.mdpi.com/article/10.3390/ani15030450/s1, Supplementary Table S1: RT-qPCR primers; Supplementary Table S2: Quality control RNA-seq data; Supplementary Table S3: Reads were aligned to the reference genome; Supplementary Table S4–S6: RNA-seq data statistics.
